# Melioidosis in Sri Lanka

**DOI:** 10.3390/tropicalmed3010022

**Published:** 2018-02-21

**Authors:** Enoka M. Corea, Aruna Dharshan de Silva, Vasanthi Thevanesam

**Affiliations:** 1 Faculty of Medicine, University of Colombo, Colombo 00800, Sri Lanka; 2 Faculty of Medicine, Kotelawala Defence University, Ratmalana 10390, Sri Lanka; dslv90@yahoo.com; 3 Faculty of Medicine, University of Peradeniya, Peradeniya 2000, Sri Lanka; vasanthithevanesam@yahoo.com

**Keywords:** melioidosis, Sri Lanka, epidemiology, *Burkholderia pseudomallei*

## Abstract

Until recently, Sri Lanka was not considered a country with endemic melioidosis. However, an increasing number of cases is being reported. National surveillance for melioidosis was instituted after 2008. A total of 250 culture-positive cases was recorded between 2006 and May 2017. Males predominated (71.6%). The age range was wide (2–92 years) reflecting a ubiquity of exposure. The majority (201/250, 80%) lived in rural areas. All provinces were affected. Case load increased during the two monsoonal periods (67%). There was representation of every population group including farmers (*n* = 44), housewives (*n* = 24), school children (*n* = 10), professionals (*n* = 5), businesspersons (*n* = 6), white-collar workers (*n* = 10) and blue-collar workers (*n* = 8). Diabetes was the predominant risk factor (*n* = 163, 65.2%). Clinical presentations included community-acquired sepsis and pneumonia, superficial and deep abscesses, and septic arthritis. Mortality was 20.4% (51/250). A majority (*n* = 212) of isolates belonged to the YLF (Yersinia-like fimbrial) clade but 38 were BTFC (*B. thailandensis*-like flagellum and chemotaxis). A total of 108 isolates was genotyped and 46 sequence types (STs) were identified, 40 being novel. It is clear that melioidosis is endemic in Sri Lanka with a wide geographic and demographic distribution. There is an urgent need to extend surveillance of melioidosis to under-resourced parts of the country and to populations at high risk.

## 1. Introduction and History of Melioidosis in the Country

Sri Lanka lies in the tropics, between 5–10° N of the equator, and within the melioidosis belt. Rice and rice flour comprise the staple diet. Paddy lands are scattered throughout the island where the predominant form of agriculture is rice farming in smallholdings, using traditional farming methods.

Sri Lanka (then Ceylon) (1927), followed British Malaya (now Malaysia and Singapore) (1921) and Cochin China (now Vietnam) (1925/6) as one of the first countries to report melioidosis, after its initial description in Rangoon, Burma (now Myanmar) in 1912 [1]. This report of fatal melioidosis in a European tea broker resident in Sri Lanka was the first from the Indian subcontinent [2] and led to the country being identified as an endemic area [3]. However, this early account was not followed by any further cases. A limited sero-epidemiological survey in hospital-associated populations in 1972 concluded that the disease was unlikely to be of major public health importance in Sri Lanka [4]. 

Reviews on the global epidemiology of melioidosis and maps depicting the known endemicity of melioidosis included Ceylon/Sri Lanka as an endemic area, based on this single case. Redfearn et al. [5] included Ceylon (Sri Lanka) in their classic map (later reproduced in a much-quoted review by Howe et al. [6]) as an area from which bacteriologically diagnosed cases of melioidosis had been reported. Leelarasamee [7] and Leelarasamee and Bovornkitti [8] listed Sri Lanka as an area where active cases of melioidosis had been seen. Dance, in 1991 [9], comprehensively reviewed the situation in Sri Lanka, identifying it as an area with sporadic isolates but, later, did not specifically refer to it in an update in 2000, probably due to a lack of subsequent cases [10]. Cheng and Currie [11] classified Sri Lanka, in their review of 2005, as belonging to an ‘area with sporadic case reports’. However, a few years later, in spite of the lack of further cases, Currie et al. [12] mapped Sri Lanka (along with the whole Indian subcontinent) as an area endemic for melioidosis and this was echoed by Wiersinga et al. in 2012 [13]. 

Seven decades after the first case of melioidosis was reported from Sri Lanka, sporadic reports began to surface of infection acquired in Sri Lanka. This included a report of brain and lung abscesses in a Belgian tourist, who had visited the country shortly before his illness [14], infection of a leg wound sustained in the 2004 Indian Ocean tsunami in an Australian man holidaying in Sri Lanka [15], and fatal septicaemia in a Sri Lankan male reported in 2006 [16]. This raised the possibility that melioidosis might be emerging in parts of Sri Lanka. As a result of a World Health Organization-supported laboratory-twinning project, additional cases were confirmed in the Central and Western Provinces in 2006 and 2008 [17,18] and surveillance for melioidosis was commenced. Recently, further cases from Sri Lanka, diagnosed locally and overseas, have been published [19,20,21,22,23,24,25,26,27,28,29,30,31,32,33,34,35].

## 2. Review of Melioidosis Cases and Presence of *B. pseudomallei* in the Country

A national network of clinical microbiology laboratories in the state and private sector was established. A case definition for surveillance and a laboratory work-up procedure for the routine culture of specimens and preliminary identification of suspected isolates was drawn up. Reference laboratory facilities for confirmation of isolates by polymerase chain reaction (PCR) for the *LPxO* gene [36] and serological testing for antibodies by the indirect haemagglutination assay (IHA) [37] were set up. A standard questionnaire for the collection of demographic, geographic and clinical data was designed. Ethics approval for the study was obtained from the Ethics Review Committee, Faculty of Medicine, University of Colombo.

Primary isolation relied on conventional culture techniques for blood and other sterile fluids, pus, and occasional soft-tissue specimens on routine culture media such as blood and MacConkey agar. Selective agar was not used for specimens from sites with normal flora so it is possible that some cases may have been missed. Suspected *B. pseudomallei* isolates were referred to the reference laboratory for confirmation by PCR. Awareness was raised, chiefly among clinical microbiologists, through lectures, presentations and publications, e-mails and personal communication. 

Between 2006 and March 2017, 250 culture-positive cases of melioidosis were identified by the surveillance programme. More than a hundred additional cases were diagnosed and treated as melioidosis on the basis of high antibody titres in the IHA test. However, this article will describe only the culture-confirmed cases. 

**Figure 1:**
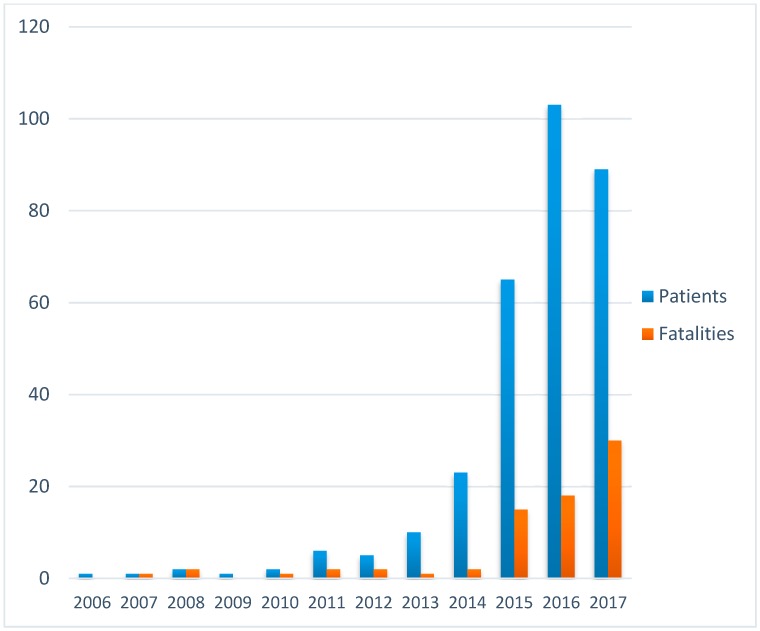
Annual incidence of cases and fatalities (2017 data only up to March).

The number of culture-positive cases increased from year to year (Figure 1). 

**Figure 2:**
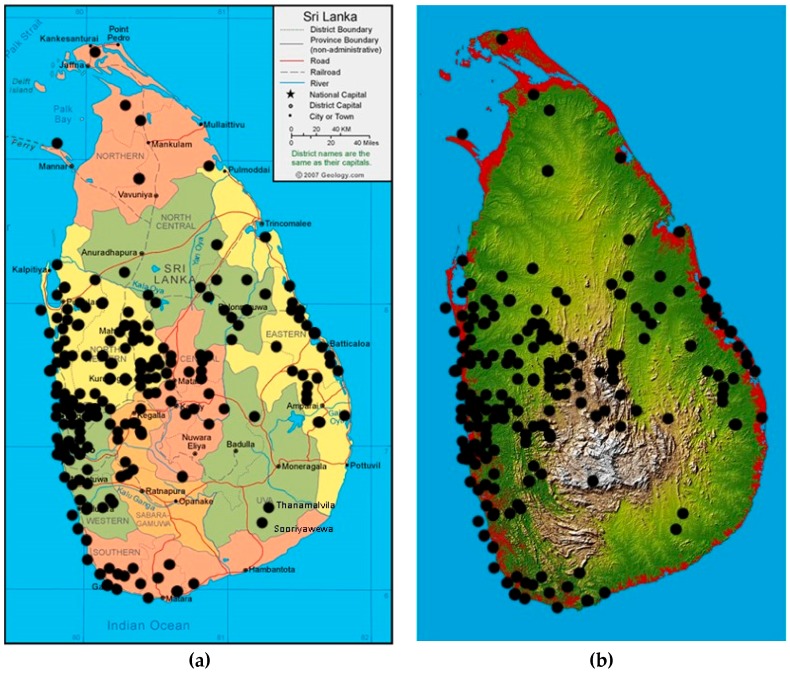
Geographical distribution of melioidosis cases in Sri Lanka. (**a**) By province. (**b**) By topography.

Melioidosis was prevalent throughout the island with all 9 provinces affected, the highest number being from the Western (*n* = 88), North-Western (*n* = 54) and Eastern (*n* = 33) provinces (Figure 2a). There were no cases at higher elevations (Figure 2b) and only a few cases in the Northern Province, where microbiology services are limited.

**Figure 3:**
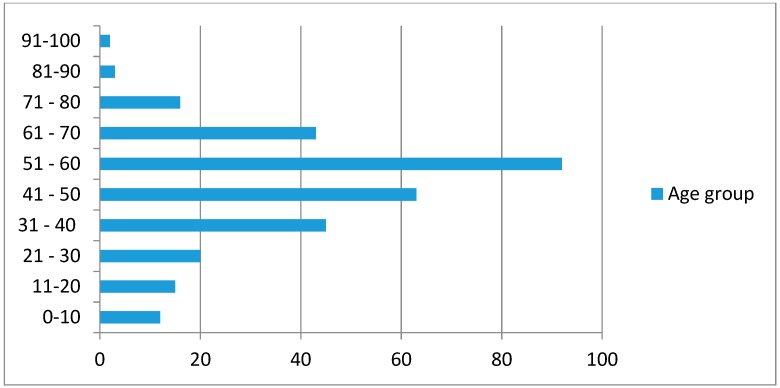
Age distribution of patients with melioidosis in Sri Lanka.

The age range of patients was wide (2–92 years), reflecting the ubiquity of soil exposure in the Sri Lankan population (Figure 3). 

The majority of patients were men (71.6%), as seen in other settings, probably reflecting occupational soil exposure in a predominantly agricultural society. Unsurprisingly, rural populations were chiefly affected (201/250, 80%). However, only 44 patients were farmers and there was representation of every population group including housewives (*n* = 24), school children (*n* = 10), professionals (including physicians and school principals) (*n* = 5), businesspersons (*n* = 6), white-collar workers including irrigation officers, technicians and clerks (*n* = 10) and blue-collar workers (*n* = 8, including labourers and construction workers). Nine patients (7%) belonged to the defence forces (army, police or civil defence) and 15 (12%) were drivers. Many gave a history of involvement in cultivation. Therefore, melioidosis in Sri Lanka seems not to be a disease limited to rice farmers but an infection related to the outdoor, agricultural, barefoot lifestyle still practised by the majority of the population. The large number of drivers, especially three-wheeler drivers and motorcyclists, in this series is intriguing and a possible explanation is exposure to dust containing *B. pseudomallei*. 

While diabetes was the predominant risk factor, found in 163 cases (65.2%), other organ disease and alcoholism were seen, and thalassemia was a significant risk in children, seen in 3 of our 10 children. Other, more unusual, predisposing causes included IgA nephropathy, dengue haemorrhagic fever, systemic lupus erythematosus (SLE) on prednisolone therapy, and lepromatous leprosy. However, melioidosis was also seen in healthy adults and children with no obvious risk factors (*n* = 30, 13.2%). 

**Figure 4:**
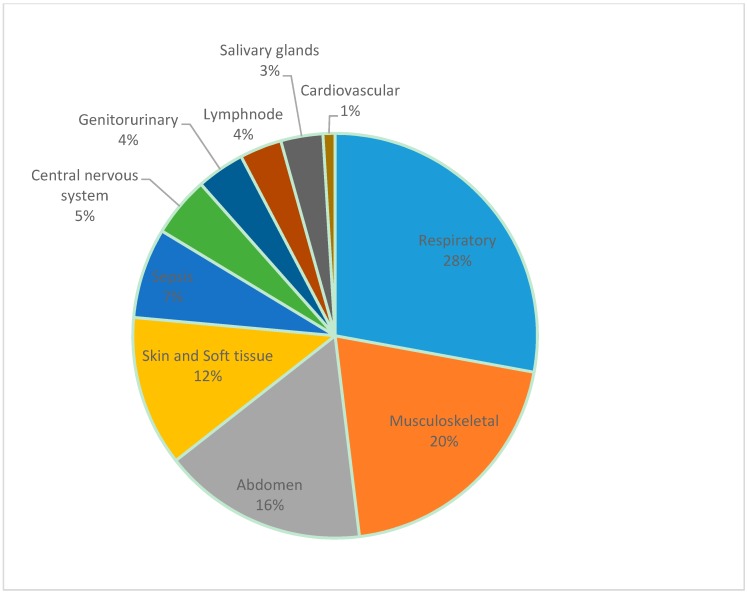
Systems affected by melioidosis in Sri Lankan patients.

As expected, clinical presentations were varied, ranging from acute sepsis to chronic abscess formation, reflecting the protean nature of this infection and manifesting in the full gamut of its clinical features (Figure 4). As in other series, lung infections predominated followed by musculoskeletal infections including septic arthritis, muscle abscesses and osteomyelitis and abdominal involvement, chiefly abscesses of the liver, spleen or psoas muscle. Skin and soft-tissue abscesses followed. While more than half the patients were blood culture-positive and septic, a few presented with septicaemia only without any obvious focus. Central nervous system infection was seen in the form of meningitis, subdural empyema, cerebral abscess, brain-stem encephalitis, transverse myelitis, Guillain Barré syndrome and status epilepticus. Genitourinary involvement was seen presenting as urinary tract infection or prostatitis. The lymph nodes and salivary glands were the site of infection in many patients. The cardiovascular system was also affected, with one patient presenting with pericardial effusion and one with endocarditis, an extremely rare presentation reported only twice previously in the literature [38,39]. Most patients had involvement of more than one organ or system.

It is of note that many patients showed involvement of the lower limbs such as septic arthritis, psoas muscle abscess and cellulitis. Septic arthritis was the primary presentation or complicated the clinical course of many patients. This may reflect bacterial entry by inoculation into the lower limbs consistent with a barefoot lifestyle.

The overall mortality was 20.4% (51/250). This compares favourably with the mortality of melioidosis in northern Australia (14%), as opposed to the mortality in Thailand (49%). However, it is far higher than the 9% mortality recorded in the latter stages of the 20-year study in Darwin [40]. Eight patients had recurrence during this period, including two who had recrudescent disease within two months of discharge whilst on eradication therapy, and four who had recurrences 6 months to 3 years after recovery (the time to recurrence was unknown in two). It is likely that some recurrences were due to non-compliance with the lengthy oral treatment required in the ‘eradication phase’. 

**Figure 5:**
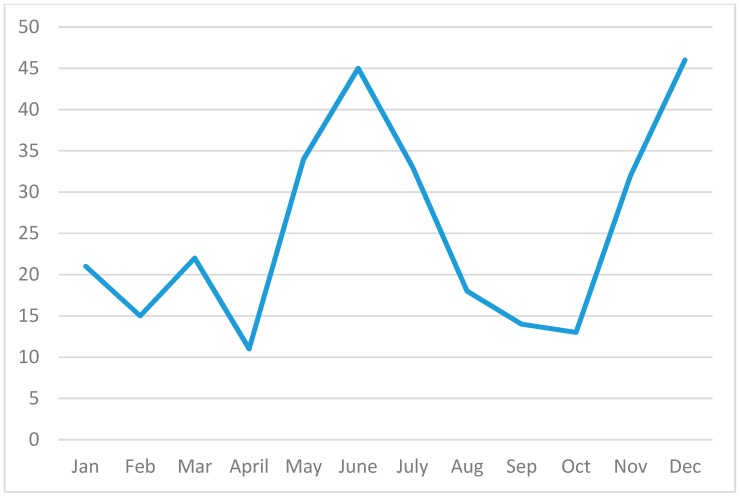
Monthly incidence of melioidosis (combined data for 2006–March 2017).

Looking more closely at the geographical distribution of this infection, melioidosis was present in the wet, intermediate, dry and even arid zones of Sri Lanka. When charting seasonal trends based on rainfall, particularly the south-western and north-eastern monsoons, it was seen that cases occur throughout the year with a trend of two peaks during the monsoons (67% of cases) (Figure 5). This is consistent with studies in many other countries that have shown increased numbers of cases during the rainy season [11]. Flooding has been associated with an increase in the incidence of melioidosis and a sharp peak was seen after torrential rainfall and flooding in May/June 2016.

While most of the cases were sporadic and unrelated to each other, there were some interesting epidemiological clusters. One was melioidosis affecting two thalassaemic siblings from Maha Oya in the Uva Province that occurred six months apart. They had probably been infected during the construction of a new house. The other was a cluster of 10 cases (with 4 deaths) that occurred in Batticaloa in the Eastern Province in October/November 2015 following heavy rains. The deaths included three female patients with severe community-acquired bronchopneumonia, suggesting acquisition via inhalation.

When the geographic data were plotted on topography and land-use maps of Sri Lanka, an infection-free area, comprising the highlands above 500 m, was noticed (Figure 2B). It seemed that while the distribution of melioidosis predictably coincided with rice-growing areas it appeared to be absent from rubber- and tea-growing regions. It is intriguing to speculate on the reason for this distribution. Is it due to the low temperatures in the hill country or different soil conditions or do the agricultural practices used in tea and rubber cultivation result in a lower risk of exposure? Further research, including soil sampling for *B. pseudomallei* in these areas, is needed to elucidate this question. 

A sero-epidemiological study was conducted in 32 blood banks distributed throughout the country on 675 blood donors using the indirect haemagglutination assay between 2011 and 2013. Antibodies to *B. pseudomallei* were quantified and the cut-off for seropositivity was set at an antibody titre of ≥40. The sero-prevalence of antibodies against *B. pseudomallei* in the study population was 7.4% (50/675). To determine demographic, geographic and other risk factors associated with positivity, binary univariate logistic regression analysis was used to generate odds ratios (OR) with 95% confidence intervals (CI). Significance was determined using a *p* value of <0.05. When the association between province and positivity was explored, donors from the North-Western Province were more likely to be positive than donors from the rest of the country, but the association did not reach statistical significance. Positivity increased progressively with age but this did not reach statistical significance. There was a significantly larger proportion of females who were antibody-positive, 17/139 (12.2%) versus 33/529 (6.2%) of males (*p* = 0.019). A significant association was noted between agriculture/gardening and positivity (*p* = 0.032) and rural location and positivity (*p* = 0.022). 

The majority of isolates (*n* = 212) belonged to the YLF (*Yersinia*-like fimbrial) gene cluster which is characteristic of South-East Asian strains. However, 38 of the bacterial strains were of the BTFC (*B. thailandensis*-like flagellum and chemotaxis) gene cluster, which is typically found in northern Australia [41]. Variation in the *bimA* gene was explored in the initial 32 isolates. While the majority of strains (*n* = 27) were *bimA*_Bp_, the variant *bimA*_Bm_, which is rarely found outside Australia, was present in 5 strains [42]. It is interesting to note that two of these patients presented with neurological melioidosis (brain-stem encephalitis and transverse myelitis, respectively), which is known to be associated with the *bimA*_Bm_ gene [43]. 

Multilocus sequence typing (MLST) [44] of 108 *B. pseudomallei* strains revealed a high diversity, with 46 different sequence types (STs) represented in the collection (Table 1). Of these, 40 were novel sequence types. This molecular epidemiology is more compatible with a bacterium that has been endemic in the country over a long period of time, even millennia, than of one that had been recently introduced. Strains from Sri Lanka cluster separately from strains from South-East Asia and Australia, suggesting separation far back in geological time [45]. Currently Sri Lanka has the largest representation (number of strains submitted) on the public MLST database of all the countries in South Asia (https://pubmlst.org/bpseudomallei/). 

In a study summarizing global evidence for the environmental presence of *B. pseudomallei* [46], where ‘definite’ presence of the bacterium was defined as the detection of *B. pseudomallei* from the environment using culture or a specific PCR, Sri Lanka was classified as a country definitely harbouring the bacterium, based on a report by Inglis et al. [17]. A preliminary study to determine the presence of *B. pseudomallei* in the soil of selected regions in Sri Lanka was undertaken in 2016. Four sites throughout the east–west axis of the island, in close proximity to locations of diagnosed cases of melioidosis, namely Nikaweratiya in the Kurunegala District of the North-Western Province, Hasalaka in the Kandy District of the Central Province and Maha Oya and Kaluwanchikudy in the Ampara and Batticaloa Districts of the Eastern Province, respectively, were studied. Soil samples were taken from rice fields and domestic gardens and subjected to molecular screening, using a quantitative real-time PCR approach with multiple *B. pseudomallei*-specific targets. *B. pseudomallei* was present in all four sites and more than 70% of all samples were *B. pseudomallei*-positive [abstract presented at World Melioidosis Congress (WMC), 2016]. 

Only one case of animal melioidosis has been reported from Sri Lanka, that of a splenic abscess in a cow that later succumbed to the infection [47]. 

## 3. Current Recommendations and Availability of Measures Against Melioidosis

An island-wide, laboratory-based, case-finding system centered on clinical microbiologists is the main surveillance system for melioidosis in Sri Lanka. Clinical microbiologists have expertise in the identification of *B. pseudomallei* in clinical specimens by routine culture. Selective media are not used. Suspected clinical isolates are referred to the reference laboratory for confirmation and a standard datasheet is completed for each culture-confirmed patient. However, it is likely that this method will fail to detect cases in areas not served by clinical microbiologists, such as large areas of the Northern Province, unless the patients are referred to other centres. 

Elucidation of the true epidemiology of melioidosis in Sri Lanka requires that it be made a notifiable disease. Steps have been taken to inform the public health authorities of the presence and extent of the infection in the country with a view to adding melioidosis to the list of notifiable diseases in Sri Lanka.

Culture-confirmed cases are treated according to standard published guidelines [48]. Government hospitals, which are well distributed throughout the country, provide treatment free of charge. Intravenous antibiotics for the acute phase and oral antibiotics for the eradication phase are available in all hospitals and there is no out-of-pocket expenditure by the patients. Intensive-care facilities are available in the larger hospitals. The moderately high case fatality rate appears to be related to late diagnosis, rather than to limitations in the availability of effective treatment.

## 4. Awareness of Melioidosis

Awareness of melioidosis among health care personnel is still inadequate and has probably contributed to the high case fatality rate. Although articles on melioidosis have appeared in the popular press, public awareness of the infection is very low, including among high-risk groups such as rice farmers and other cultivators. Island-wide awareness programmes to alert clinicians and public health authorities to the presence and presentation of melioidosis are needed.

## 5. Major Achievements

Although sporadic cases of melioidosis have been described previously in Sri Lanka, this study establishes, conclusively, that melioidosis is endemic in Sri Lanka, with a wide geographic and demographic distribution. The absence of melioidosis in the high hills (>500 m above sea level) is noted. This may be due to the low temperatures seen in these areas or the different agricultural practices used in these regions, where tea is the main crop, as opposed to rice as in most other areas of the country. The seasonal nature of the incidence of melioidosis, with an increase during the monsoons, is demonstrated. 

The demographic profile of melioidosis in Sri Lanka is described for the first time and is broadly similar to countries with similar climate and agriculture. However, the wide distribution of cases in all strata of society and all grades of occupation is noted. The proposition that the rural, outdoor, agricultural, barefoot lifestyle of the majority of the population poses a general risk of acquisition of melioidosis is postulated. Individuals in the defence forces and construction workers have been confirmed to be at risk, as shown previously, and new risk groups such as housewives and drivers have been identified. 

The high rates of co-morbidity in patients in Sri Lanka, compared to other endemic areas such as Thailand and Vietnam, is likely to reflect lower exposure to *B. pseudomallei*, probably due to lower bacterial loads in the environment, so that clinical disease develops mainly in highly susceptible populations. 

A higher rate of bacteraemic cases and a lower rate of localized cases were seen in this series and may be an artefact of blood culture-positive patients being investigated more thoroughly. The case profile resembled that described previously in other case series. Neurological melioidosis, which has previously been described mainly in northern Australia, was seen. Musculoskeletal melioidosis was more common and may be the consequence of a barefoot lifestyle. 

Genotyping of bacterial strains revealed a high diversity of sequence types, widely distributed throughout the island, confirming that the bacterium is endemic to the country. Strains from Sri Lanka clustered together and separately from the south-east Asian and Australian strains, showing regional specificity.

## 6. Current and Future Challenges

Increased construction and building activities in Sri Lanka may place construction workers, engineers and the general public at risk. Small-scale farming, gardening and horticulture have become popular for income generation and may increase exposure to this soil bacterium. Climate change has led to an increase in natural disasters, such as storms, landslides and flooding, that expose even healthy persons to high doses and unusual routes of transmission. 

Exposure-mitigating practices, such as use of mechanical agriculture and protective clothing when coming into contact with mud and water, may reduce infection with *B. pseudomallei.* However, this may not be feasible due to their lack of acceptability and adverse socioeconomic impact. The use of doxycycline in farmers for leptospirosis prophylaxis may also protect against the acquisition of melioidosis. The evidence base for such strategies needs to be studied. Other ‘at risk’ occupations such as manual labourers, irrigation, water supply, construction, mining and road repair workers may also need to adopt similar preventive measures, when required. Preventive strategies should include more effective community-based diabetes prevention, detection and control that will reduce the risk of clinical disease. Recrudescent melioidosis should be included in the agenda of migrant health issues, including that of the diaspora that left Sri Lanka following civil disturbances over the past few decades. 

Sero-surveillance at the community level may serve to obtain a true reflection of the extent of exposure to *B. pseudomallei* in the community. Targeted sero-surveillance in high-risk groups such as rice farmers, defence personnel and diabetics may help to quantify the risk of clinical disease in these individuals. Studies of the development of antibodies to *B. pseudomallei* during childhood and estimation of the intensity of exposure and incidence of melioidosis in children by IHA testing of a large cohort of children, with each year equally represented, would also add to knowledge of the extent and timing of bacterial exposure.

Soil sampling to detect and quantify the presence of *B. pseudomallei* in soil in Sri Lanka and mapping of the distribution of environmental *B. pseudomallei* will enable identification of high-risk geographic regions where heightened case detection can be implemented. The intriguing lack of infection in the high hills should be explored by soil sampling at different altitudes and from different agricultural settings. 

Genotyping of further strains of *B. pseudomallei* will be useful to confirm the diversity of sequence types in Sri Lanka and to explore any associations of genotype with geographical distribution, clinical presentation or virulence and any genetic difference between environmental and clinical strains. The presence of the BTFC and *BimA*_Bm_ variants in significant numbers in Sri Lanka will have implications for the geographical origins and evolution of the bacterium in geological time. Whole-genome sequencing will allow further discrimination of south-Asian strains from those in Oceania and south-east Asia and help deduce evolutionary relatedness between isolates from these regions. 

The One Health concept is founded on the premise that the health of the environment, of animals and humans are inextricably linked. Melioidosis, caused by a soil saprophyte and infecting both animals and humans through soil and water exposure is the ideal candidate infection for the One Health approach to disease mitigation and health promotion. Integrating local, national and global workers in multidisciplinary teams to research this infection with a view to reducing its impact on human and animal populations is essential to this endeavour. Convening such a platform for the south-Asian region, which probably bears the highest burden of melioidosis but where the disease is currently grossly under-diagnosed, is an urgent imperative.

## Figures and Tables

**Table 1:** Sequence types (ST) of *B. pseudomallei* strains from Sri Lanka.

ST	No	ST	No	ST	No	ST	No	ST	No	ST	No
13	2	590		944		1139	3	1147	3	1435	2
132		594	5	1132	8	1140	5	1148		1436	
194		598		1133		1141		1152	2	1437	
202		615		1134		1142	2	1179		1438	
293		655		1135	9	1143	2	1314		1439	
308		733		1136	8	1144		1364		1442	
338		867		1137	18	1145		1413			
474		912	2	1138		1146		1434	6		
